# Supplementary material for: A set of multi-entry identification keys to African frugivorous flies (Diptera, Tephritidae)
Source: Zookeys. 2014 Jul 24;(428):97–108. doi: 10.3897/zookeys.428.7366 (PMC4143993; doi:10.3897/zookeys.428.7366)
Supplement: Supplementary material 4 — Key to Capparimyia [file zookeys-428-097-s004.zip › SF4_ZooKeys_key to Capparimyia/lucid_player/help/help/pruning_the_feature_tree.htm]

Pruning the feature tree - The Prune Redundants function


# Pruning the feature tree - The Prune Redundants function

After a number of features have been chosen and the *Entities
Remaining* list has been substantially reduced, some features and
states may be redundant for the identification.

A state is considered redundant if it applies to none of the entities
remaining, or to all of the entities remaining (choosing such a state
will either leave no entities or remove no entities, so it is useless
to answer). A feature is redundant if all of its states are redundant.

The *Prune Redundants* function
will scan the features in *Features Available*
and remove any redundant features and/or states for the remaining entities.
This may be useful to �clean up� the list of features, to make it less
likely that a choice will be useless.

**Note** that there is
one circumstance in which apparently redundant states may be left in after
pruning. Consider a 2-state feature, one state of which would remove some
remaining taxa, the other of which would remove none. The state that removes
no taxa is not logically redundant in this case, and would not be removed
since removing it would leave a character with only one state.

To activate the *Prune Redundants*
function, choose *Prune Redundants*
from the *Features* menu, or click
the *Prune* button  on the toolbar.

*Prune Redundants* can be automated,
so that it is recalculated after each feature is addressed. Choose the
option *Automate*>*AutoPrune*
from the *Features* menu. If you
need to choose several states from one feature when *AutoPrune*
is on, hold down the *Control*
key when choosing the states � *AutoPrune*
will be paused until the *Control*
key is released.
